# High Origin of the Medial Circumflex Femoral Artery From the External Iliac Artery in Common Trunk With the Inferior Epigastric Artery

**DOI:** 10.7759/cureus.66508

**Published:** 2024-08-09

**Authors:** Albert Gradev, Vasil Iliev, Lazar Jelev

**Affiliations:** 1 Department of Anatomy, Histology and Embryology, Medical University-Sofia, Sofia, BGR

**Keywords:** external iliac artery, cadaver, inferior epigastric artery, medial circumflex femoral artery, anatomy variation

## Abstract

The medial circumflex femoral artery contributes to the blood supply of the adductor muscles, hip joint, and femoral head. Its variations are common and important in the surgical field, as its damage can cause femoral head necrosis. Most commonly, the variations include different origin patterns from the femoral artery or its branches.

Here we report a very rare variation of suprainguinal origin of the medial circumflex femoral artery from the external iliac artery in the common trunk with the inferior epigastric artery. Because of the rarity, such an arterial variation not commonly suspected during open or laparoscopic surgery may result in devastating consequences.

## Introduction

The medial circumflex femoral artery (MCFA) is the major nourishing artery of the adductor thigh muscles, sending branches to the hip joint and supplying the femoral head. Its most common origin is from the medial aspect of the deep femoral artery, but sometimes it can originate directly from the femoral artery [1] or another source [2]. The variations of the origin and course of the MCFA are important for open surgical procedures within the femoral triangle and inguinal region. Quite rare, the MCFA can arise from the stem artery superior to the inguinal ligament (suprainguinal origin), and this origin may cause problems during hernia repair surgery. This unsuspected high arterial origin increases the risk of iatrogenic injury, which could lead to different complications, including femoral head necrosis.

## Case presentation

**Figure 1:**
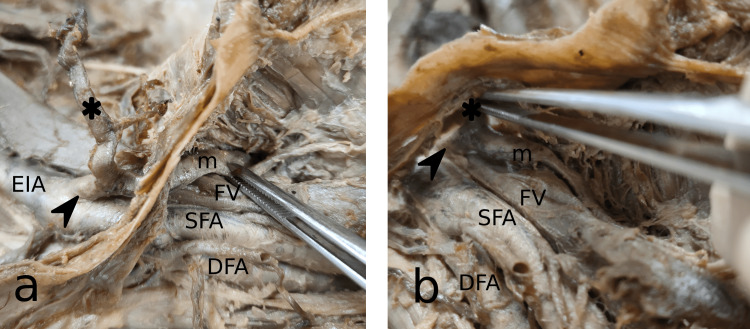
(a) Anterolateral view of the dissected right lower limb at the level of the inguinal ligament. (b) Anteroinferior view of the dissected right lower limb below the level of the inguinal ligament within the femoral triangle EIA: External iliac artery; SFA: superficial femoral artery; DFA: deep femoral artery; FV: femoral vein; m: medial circumflex femoral artery; arrowhead: common trunk; asterisk: inferior epigastric artery

The reported findings were observed during routine student dissections of the right lower limb of an adult formalin-fixed 74-year-old female cadaver of Caucasian descent. All dissections took place at the Department of Anatomy, Histology and Embryology, Medical University of Sofia. We discovered a rare unilateral variation of the origin of MCFA (Figure 1a). After removing the parietal peritoneum in the right iliac region, we found the inferior epigastric artery (IEA) (external diameter 3.5mm) in its usual anatomical place at the lateral boundary of the Hesselbach triangle. After precise dissection proximally, we revealed a common trunk of the latter and another mid-sized artery, which turned downward to the anterior femoral region. The common arterial trunk (external diameter 5 mm) originated from the anterior surface of the external iliac artery at a distance of 12 mm above the inguinal ligament. It courses medially, downward, and anteriorly and just above the superior border of the inguinal ligament bifurcated into the IEA and the aberrant femoral artery, which complete dissection within the femoral triangle revealed an MCFA of high suprainguinal origin. From the bifurcation point, the aberrant MCFA (external diameter 4mm) passed underneath the inguinal ligament through the lacuna vasorum in a direction lateral to medial, crossing in front of the external iliac-femoral vein and reaching the posterior border of the pectineus muscle (Figure 1b). Further distally, it continues between pectineus and iliopsoas having a usual distal course. In the femoral triangle, it sent branches to the pectineus muscle and gave rise to one deep pudendal artery. Additionally, we found the origin of the deep femoral artery to be very high - 13mm below the inguinal ligament. The deep femoral artery in this case gave the origin of the lateral circumflex femoral artery only.

## Discussion

**Figure 2:**
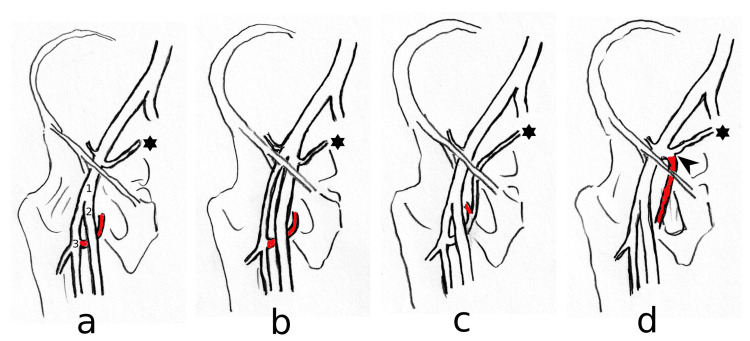
Schematic drawing representing the different sites of origin of the MCFA (highlighted in red). The figure is author's own creation. (a) MCFA originates at its usual site from the deep femoral artery; (b) MCFA originates from the deep femoral artery with coincidence of high bifurcation of the femoral artery; (c) origin of the MCFA with the inferior epigastric artery in a common stem from the deep femoral artery; (d) origin of the MCFA with the inferior epigastric artery in a common stem from the external iliac artery as presented in our case asterisk: Inferior epigastric artery, arrowhead: common stem of the MCFA and inferior epigastric artery from the external iliac artery; 1: femoral artery; 2: superficial femoral artery; 3: deep femoral artery; MCFA: medial circumflex femoral artery

The reported high suprainguinal origin of MCFA seems to be quite rare arterial variation with only a few cases reported in the pertinent literature. We made schematic drawing to represent the different most frequent abnormal origins of the MCFA (Figure 2). In the most detailed book on arterial variations, Adachi [3] reported five such cases: only two by his own dissections, and one case by Thompson, Giacomini, and Bruns. Adachi’s first case was similar to ours but on the left side. In his second case, in a male cadaver, on the right side, the common stem was crossed by the ductus deferens, and the bifurcation into IEA and MCFA was more superiorly placed, so the MCFA passes along the medial side of the femoral vein for a short distance. After further literature review, we found that Thompson [4] described in fact two cases in which the IEA and MCFA originate in the common trunk above the inguinal ligament - approximately 8 mm in the first case and 6mm in the second case. In the first case, the artery passes through the femoral canal, and in the second - through lacuna vasorum. He also cited different authors, but only the case of Dr. John Reid seems similar to ours. Giacomini [5] described such a case in which the IEA and MCFA originate as a common trunk in the abdominal cavity, and then the MCFA descends anterior to the femoral vein below the inguinal ligament. A similar case is also reported by Javadnia [6]. Bergman [2] in his Encyclopedia cites Adachi [3] and Fisher [7]. We can conclude that there are described no more than nine similar cases in the literature, but only two like ours - one from Adachi, and one from Thompson. We can conclude that this is a very rare variation, seen not more than 10 times (including our report) during the past two centuries. We found in the literature also several cases with the common trunk of MCFA and IEA, sometimes with the obturator artery involved [3,8-13]. In the cases in which the obturator artery is involved in the common trunk, the trunk originates from the external iliac artery. In other cases, it originates from the femoral artery below the inguinal ligament.

Documented here high bifurcation of the common femoral artery is not very rare with an estimated frequency of 26% and 8% for high and very high origin [14] measured by relation to the femoral head in angiography. If we estimate the mean distance of the bifurcation from the inguinal ligament, it will be approximately 3.2-5.3cm [15]. After the bifurcation, the arteries can have different patterns of branching, ours is the so-called truncus profundocircumflexus lateralis [3], with MCFA origin from another source. In the most common cases, the origin is directly from the femoral artery [1]. Mostly, in documented high bifurcations, there is no correlation with the high origin of MCFA.

Nowadays with widely used angiographic studies, such a high origin of MCFA was not yet documented [14,16], also it is not yet documented in some large cadaveric studies from different populations [17-19].

## Conclusions

The variation reported by us is considered extremely rare. Despite its rarity, it could have a significant clinical impact. The abnormal origin of MCFA could increase the risk of damage to the artery during extraperitoneal dissection for laparoscopic hernia repair. It also can be damaged during femoral hernia surgery. The artery can compress the femoral vein behind the inguinal ligament, which can cause venous blood retention. Damage to the artery can cause femoral neck necrosis.
